# Michigan State Board of Health

**Published:** 1880-08

**Authors:** 


### ARTICLE VIII.

THE MICHIGAN STATE BOARD OF HEALTH.

The regular quarterly meeting of this board was held at their rooms in the State Capitol, at Lansing, on Tuesday, July 13th, commencing at 9 o'clock a. m. The following members were present: Dr. R. C. Kedzie, president, of Lansing; Rev. Dr. D. C. Jacokes, of Pontiac; Dr. Henry F. Lyster, of Detroit; Dr. J. H. Kellogg, of Battle Creek and Dr. Henry B. Baker, secretary.

Dr. Lyster called the attention of the board to syphilis, a disease to which but little attention was paid by sanitarians, but which causes much sickness and many deaths in this State. He was requested to prepare a paper on the subject and present it at the next meeting of the board.

The resignation of Dr. H. O. Hitchcock, of Kalamazoo, as a member of the board, and the appointment of Prof. E. A. Strong, of Grand Rapids, by the governor, were announced and the following resolutions were adopted:

*Resolved*, That in the retirement of Dr. Hitchcock from membership in this board, the board loses one of its most efficient and distinguished members.

*Resolved*, That the individual members of the board regret the personal separation thereby entailed, and extend to the retiring member their best wishes for his continued prosperity.

A letter from Dr. Hitchcock commended very highly his successor, Prof. Strong, as also did other members.

BOARD OF HEALTH IN DETROIT.

The secretary presented a communication from F. G. Russell, city attorney of Detroit, suggesting that the state board address a letter to the mayor and aldermen of that city, recommending the organization of a board of health, and the appointment of a health officer.

Dr. Lyster said there was no way of getting reliable statistics relative to sickness and mortality in Detroit. The record of interments is the only source of information, and is not reliable, as the reports of the city clerk are voluntary, and there are many interments (especially of Israelites) outside the city. The old board of health was not efficient because unwieldy, but the "sanitary squad," of the police force, does some efficient work in enforcing the ordinances relative to garbage, etc. The city police, however, oppose the appointment of a health officer, fearing it will interfere with the work of their "sanitary squad." It was suggested that perhaps the people of Detroit did not wish the real facts relative to sickness and death disclosed. Drs. Lyster and Baker were appointed to prepare a plan for a board of health in that city and endeavor to secure its adoption.

SICKNESS AND PAUPERISM.

A communication was presented from Hon. H. W. Lord, secretary of the state board of corrections and charities, relative to pauperism as a result of sickness. After some discussion relative to the amount of pauperism caused by sickness, and the extent of the field over which a study into the subject should reach, a committee was appointed to investigate the subject, to be known as "the committee on the relations of preventable sickness to taxation," with Dr. J. H. Kellogg as chairman.

SANITARY SCIENCE EXAMINATIONS.

The remainder of the forenoon session was principally occupied with routine work and the perfection of details for examining and marking the standing of candidates in the examinations in sanitary science inaugurated the following day, and which require: "The replies on each set of topics shall be marked on a scale of 10, and an average standing of 70 per cent. on all topics shall be necessary in order to pass the applicant." One who successfully passes the examination receives a certificate that he is considered qualified to act as health officer of any township, city or village in Michigan.

A paper on "unsanitary conditions in our schools," by G. E. Corbin, M. D., of St. Johns, was read. The paper consisted of details of overcrowding, bad ventilation and the sickness resulting therefrom, which came under his personal observation. The paper will be published in the report for 1880.

Two valuable papers by A. W. Nicholson, M. D., of Otisville, were presented. One was on "ozone," and contains details of numerous experiments, and one on "periodic fever," containing detailed records of cases and coincident meteorological conditions. The papers were accepted with thanks, and ordered printed in the annual report for 1880.

### SANITARY CONVENTIONS.

The secretary reported that he had edited and prepared for publication the proceedings, etc. of the sanitary conventions held at Detroit and Grand Rapids during the past winter, and the copy was in the hands of the printer.

### ADULTERATIONS OF FOODS.

Dr. Kedzie said he had received a request from gentlemen in Chicago to enter upon an investigation of adulterations of foods, and had replied that the board had no funds. He stated that the adulteration of sugar with glucose was increasing rapidly, and was being done more skilfully; that adulteration with pure glucose did not endanger health, but the sugar was not so sweet. The manufactured glucose, however, was unhealthful to take into the stomach, because of poisonous substances which are always associated with it. Dr. Lyster said a prominent candy dealer had informed him that all candies, except rock candies, were composed in part of glucose. Dr. Kedzie said nearly all syrups were made from glucose.

The board performed a large amount of routine work, such as auditing of bills, and adjourned until October 12, 1880.

